# A Web Service for Biomedical Term Look-Up

**DOI:** 10.1002/cfg.459

**Published:** 2005

**Authors:** Henk Harkema, Ian Roberts, Rob Gaizauskas, Mark Hepple

**Affiliations:** 1 Department of Computer Science University of Sheffield Sheffield UK; 2 Dept of Computer Science University of Sheffield Regent Court 211 Portobello Street Sheffield S1 4DP UK

## Abstract

Recent years have seen a huge increase in the amount of biomedical information that is available in electronic format. Consequently, for biomedical researchers wishing to relate their experimental results to relevant data lurking somewhere within this expanding universe of on-line information, the ability to access and navigate biomedical information sources in an efficient manner has become increasingly important. Natural language and text processing techniques can facilitate this task by making the information contained in textual resources such as MEDLINE more readily accessible and amenable to computational processing. Names of biological entities such as genes and proteins provide critical links between different biomedical information sources and researchers' experimental data. Therefore, automatic identification and classification of these terms in text is an essential capability of any natural language processing system aimed at managing the wealth of biomedical information that is available electronically. To support term recognition in the biomedical domain, we have developed Termino, a large-scale terminological resource for text processing applications, which has two main components: first, a database into which very large numbers of terms can be loaded from resources such as UMLS, and stored together with various kinds of relevant information; second, a finite state recognizer, for fast and efficient identification and mark-up of terms within text. Since many biomedical applications require this functionality, we have made Termino available to the community as a web service, which allows for its integration into larger applications as a remotely located component, accessed through a standardized interface over the web.


**Comparative and Functional Genomics**
*Comp Funct Genom* 2005; **6**: 86–93.
Published online in Wiley InterScience (www.interscience.wiley.com). **DOI:** 10.1002/cfg.459

**Conference Paper**

### A web service for biomedical term look-up

Henk Harkema*, Ian Roberts, Rob Gaizauskas and Mark Hepple
*Department of Computer Science, University of Sheffield, Sheffield, UK*

**Correspondence to:*
*Henk Harkema, Dept of Computer Science, University of Sheffield, Regent Court, 211 Portobello Street, Sheffield S1 4DP, UK.*
*E-mail: harkema@dcs.shef.ac.uk*

Received: 17 December 2004
Accepted: 7 January 2005

### Abstract

**Recent years have seen a huge increase in the amount of biomedical information that is available in electronic format. Consequently, for biomedical researchers wishing to relate their experimental results to relevant data lurking somewhere within this expanding universe of on-line information, the ability to access and navigate biomedical information sources in an efficient manner has become increasingly important. Natural language and text processing techniques can facilitate this task by making the information contained in textual resources such as MEDLINE more readily accessible and amenable to computational processing. Names of biological entities such as genes and proteins provide critical links between different biomedical information sources and researchers' experimental data. Therefore, automatic identification and classification of these terms in text is an essential capability of any natural language processing system aimed at managing the wealth of biomedical information that is available electronically. To support term recognition in the biomedical domain, we have developed Termino, a large-scale terminological resource for text processing applications, which has two main components: first, a database into which very large numbers of terms can be loaded from resources such as UMLS, and stored together with various kinds of relevant information; second, a finite state recognizer, for fast and efficient identification and mark-up of terms within text. Since many biomedical applications require this functionality, we have made Termino available to the community as a web service, which allows for its integration into larger applications as a remotely located component, accessed through a standardized interface over the web. Copyright © 2005 John Wiley & Sons, Ltd.**

**Keywords:** natural language processing; terminology; biomedical research; information storage and retrieval; software design; internet

### Introduction

Recent years have seen a huge increase in the amount of biomedical information that is available in electronic format. Some of this information resides in structured databases and ontologies such as OMIM (Online Mendelian Inheritance in Man [20]) and the Gene Ontology [26]. On-line biomedical literature collections, e.g. MEDLINE [22], form another important source of biomedical information. These latter collections provide information in an unstructured, textual format.

Consequently, for any biomedical researcher wishing to relate their experimental results to relevant data lurking somewhere within this expanding universe of on-line information, the ability to access and navigate biomedical information sources in an efficient manner is increasingly important. Natural language and text processing techniques can facilitate this task by making the information contained in textual resources more readily accessible and amenable to computational processing. These techniques can be applied to automatically capture, structure and further process the semantic content of journal abstracts and papers, where 'semantic content' can be understood at various levels of abstraction, ranging from the set of domain-specific terms that occur in a document to structured descriptions of the biomedical entities and the relationships between them that are

Copyright © 2005 John Wiley & Sons, Ltd.

expressed in a document (e.g. [4,15,25]). Because of their automatic nature and the structured format of the results, these techniques provide rapid and efficient computational access to the contents of very large numbers of documents.

With regard to biomedical text processing, Hirschman *et al.* [18] explicitly note that the names of biological entities such as genes and proteins provide the critical links across the different biomedical information sources and researchers' experimental data. Therefore, automatic identification and classification of these terms in text is an essential capability of any natural language processing system aimed at managing the wealth of biomedical information that is available electronically. To support term recognition in the biomedical domain, we have developed Termino, a large-scale terminological resource for text processing applications [16,17]. It includes a flexible, extensible relational database which stores large numbers of terms together with complex, heterogeneous information about these terms. The system comes equipped with a compiler for generating finite state term recognizers from the contents of the database.

Since term recognition is of paramount importance to biomedical information processing, most, if not all, applications in this domain require access to terminological knowledge. For this reason we are making Termino available to the biomedical text mining community as a web service. This service provides public, standardized access to Termino over the web, allowing integration of this resource into applications developed by other research groups as a remotely located component. The web service delivers lexical look-up functionality: given a text, the service will return a version of the text in which occurrences of terms are identified and marked up with information from Termino. We have also implemented a web browser-based interface to the web service, which provides potential users with a simple way of exploring the utility of the web service.

In the remainder of this paper we give a brief description of Termino and outline how we have implemented web service access to this resource.

### Architecture and functionality of Termino

Termino is a large-scale terminological resource for text processing applications. It includes a flexible, extensible relational database, which stores large numbers of terms together with complex, heterogeneous information about these terms, including information of a morphosyntactic nature, such as part of speech and morphological class; information of a semantic nature, such as quasi-logical form and links to concepts in ontologies; and provenance information, such as the sources of the information in the database. The design of the database also allows for links to connect synonyms and morphological and orthographic variants to one another, and links to connect abbreviations and acronyms to their full forms.

To ensure fast term look-up with Termino's potentially vast terminological database, the system comes equipped with a compiler for generating finite state machines from the strings in the terminological database. To recognize terms in text, the recognizer is run starting at each position, i.e. token, in the text. It will determine very quickly whether there are any strings in the database beginning at this point and, if so, with what information in the database these strings are associated. This set-up, in which term recognizers are compiled from the contents of the terminological database, turns Termino into a general terminological resource that is not restricted to any single domain or application. The database can be loaded with terms from multiple domains and compilation can be restricted to particular subsets of strings in the database by selection, e.g. based on their source or other characteristics. In this way one can produce term recognizers that are tailored towards specific domains or specific applications within domains.

The contents of Termino's database are imported from existing, outside knowledge sources, such as the HUGO Gene Nomenclature database [28] and the Metathesaurus of the Unified Medical Language System (UMLS [19,23]). Contents can also be obtained through automatic term induction from on-line text corpora, e.g. MEDLINE citations. Termino thus enables uniform access to terminological information aggregated across many sources, without the need for multiple, source-specific terminological components within a text processing system. Term look-up in Termino provides immediate entry points into a variety of outside ontologies and other knowledge sources, making the information in these sources available to processing steps subsequent to term recognition. (This advantage does not extend to terms that are added to Termino through

Copyright © 2005 John Wiley & Sons, Ltd. 

term induction). For example, using a recognizer compiled to include terms from the HUGO and OMIM databases, Termino will return the HUGO and OMIM identifiers for the gene names it recognizes in a text. These identifiers give access to the information stored in these databases about the gene, including alternative names, gene map locus, related disorders and references to relevant papers.

Termino is designed to be the first component in a multi-component term processing system. Thus, term look-up as performed by Termino is not the end point of term processing. Look-up might return multiple matching terms for a given string, or for overlapping strings, and subsequent processes may apply to filter these alternatives down to the single option that seems most likely to be correct in the given context. Furthermore, more flexible processes of term recognition might apply over the results of look-up, e.g. a term *grammar* can be provided for a given domain, allowing longer terms to be built from shorter terms that have been identified by term look-up (e.g. [12]).

Termino's database currently contains well over 400,000 terms. For further details about the design and implementation of Termino the reader is referred to [16] and [17].

### Term look-up web service

Since term recognition is an important aspect of biomedical text processing, we think it would be useful to share Termino with the wider biomedical text mining research community. We have decided to do so by making Termino available in the form of a web service.

In the following sections we will give a brief overview of web services in general and point to a few bioinformatics and natural language-processing web service applications, describe the functionality of Termino as a web service, and discuss some issues concerning the implementation of this particular web service.

### Web services

A web service (e.g. [10,27]) provides access to data or processing resources on the web through standard internet protocols. Typically, using a web service proceeds according to the following scenario. First, a client looking for a particular service will consult a global registry of web services to find a host providing the desired service. The registry describes, in a standard manner, the functionality of a given web service and the way in which the web service can be accessed. Next, based on the information found in the registry, the client will select a particular web service and use it. Using a web service involves the transmission of standardized messages for invoking the service and receiving back the results of the invocation. The high degree of standardization of all aspects of the web service paradigm — discovery, description and usage — facilitates simple access across different software platforms to any resource packaged as a web service and enables easy integration of such a resource into distributed applications. Web services are thus an effective means for sharing resources between research groups. Their use promotes collaboration and prevents duplication of efforts spent on developing resources [7].

Further advantages of publishing Termino as a web service rather than releasing the resource as a downloadable software package include:

- Users can access and integrate Termino into their applications without having to download and install the heterogeneous set of software components comprised in Termino.
- Since the code and the data are located at our site, users do not have to download and install a new version of Termino each time the code is updated or the terminological database is expanded.
- Web service access to Termino allows us to monitor its usage, providing us with a measure of its actual utility.

Bioinformatics experiments produce large amounts of data which are of value not only to the researchers conducting the experiment but to others in the field as well. In order to share the data within the research community, it is deposited in public repositories, many of which are now programmatically accessible via web services (e.g. [9]). Similarly, many biomedical processing resources are also available through a web service interface (e.g. [21]).

There is also growing interest in the use of web services for natural language processing applications. For example, Biemann *et al.* [3] discuss the deployment of web service technology in

Copyright © 2005 John Wiley & Sons, Ltd. 

the domain of corpus processing. Dalli *et al.* [7] describe a general web service-based architecture for language resources and present details about the implementation of two web service applications built according to this architecture. Curran [6] shows how web services can be used to provide interfaces to a high-performance infrastructure for natural language processing. Quasthoff and Wolff [24] give an example of a web service for dictionary look-up and terminology extraction. Gaizauskas *et al.* [11] describe an application in which web services are used to incorporate text mining capabilities into a workflow environment for supporting scientific discovery in bioinformatics. If, as expected, web services become the dominant paradigm for communication within distributed systems, more and more natural language processing resources will be made available as web services.

### Functionality

The web service built on top of Termino has been designed to implement a lexical look-up service: given a text, the service returns a version of the text in which term occurrences are identified and marked up with information from the Termino database. A request to the web service includes the text to be processed or its URL, so that the web service can download the text. Furthermore, in the service request the user can indicate the classes of terms that are to be tagged in the text, e.g. 'gene', 'protein', 'body part', etc. These classes are organized into a simple ontology, enabling the user to choose a set of classes matching the semantic granularity of the intended application. The selection of term classes determines the set of recognizers compiled from Termino's database that will be run over the input text. In the current prototype, we offer a set of pre-compiled recognizers from which the user can make a choice, covering the term classes 'gene', 'disease or syndrome', and 'human protein'. The terms in the first class are imported from the HUGO and OMIM databases, the terms in the second class come from UMLS, and the terms in the third class originate from the European Bioinformatics Institute's Gene Ontology Annotation project [8]. At a later stage, we may consider a scenario in which recognizers will be compiled at request time so that users can dynamically ask for mark-up of arbitrary term classes.

The response to a request to the web service is a text in XML format in which occurrences of terms are labelled with the term classes they belong to and are annotated with additional information from Termino's database. As Termino provides a lexical look-up service rather than full term identification and classification, terms that are assigned multiple classes are not disambiguated. Additional information for a term may include, for example, a UMLS unique concept identifier, HUGO and OMIM database identifiers and assignments to nodes in the Gene Ontology. In the current implementation of the web service, all information in the Termino database found for a term is returned; in the next version the user will be able to ask for only specific kinds of information from the database. The next version of the web service will also draw on the synonymy information stored in Termino to supply synonym class identifiers for terms. These identifiers may be used to determine which terms in a text are synonymous.

It is possible that a text will contain terms with overlapping spans, e.g. in the phrase *middle ear infection*, the sequence *middle ear* may be recognized as a 'body part, organ, or organ component' and the sequence *ear infection* may be marked-up as belonging to the class 'disease or syndrome'. (It is hard to find an example of overlapping terms where the terms are drawn from the classes 'gene', 'human protein' and 'disease or syndrome'. Therefore the example given involves the term classes 'body part, organ, or organ component' and 'disease or syndrome', both of which contain terms from UMLS). Such overlapping terms cannot be represented using standard in-line XML mark-up. One solution to this problem is to select only one of the overlapping terms for mark-up. However, the decision about which term to keep and which term to discard is generally application-dependent and should therefore not be made by the term look-up service. We address this problem by adopting a slightly more complex XML encoding, which combines characteristics of in-line and stand-off mark-up, and which can represent overlapping terms. This approach allows users to choose for themselves amongst overlapping term possibilities.

The XML encoding of terms involves inserting empty `Node` elements into the input text, embracing portions of the text that are terms. Thus, the phrase *middle ear infection* will have nodes

Copyright © 2005 John Wiley & Sons, Ltd.

inserted between adjacent tokens, including spaces, as follows:

```
<Node id='0'/>middle
<Node id='1'/>ear <Node
id='2'/> <Node id='3'/> infection
<Node id='4'/>
```

The terms in this phrase can now be annotated as follows:

```
<Body_part startNode='0' endNode='2'>
                    <cui>C0013455</cui>
</Body_part>
<Disease startNode='0' endNode='4'>
                    <cui>C0029882</cui>
</Disease>
<Body_part startNode='1' endNode='2'>
                    <cui>C0013443</cui>
</Body_part>
<Disease startNode='1' endNode='4'>
                    <cui>C0699744</cui>
</Disease>
<Disease startNode='3' endNode='4'>
                    <cui>C0021311</cui>
</Disease>
```

These annotations state, among other things, that the text between nodes 1 and 4, i.e. *ear infection*, is a term belonging to the class 'disease or syndrome' and that in UMLS this term is associated with the unique concept identifier (CUI) C0699744.

Figure 1 shows the complete XML document returned by the Termino web service when the recognizers for the term classes 'gene' and 'disease or syndrome' are turned on and the input is a document containing the single sentence *Since mutations in the gamma-crystallin encoding CRYG genes have previously been demonstrated to be the most frequent reason for isolated congenital cataracts, all four active CRYG genes have been sequenced.* The `Text` element contains the text of the input document into which `Node` elements have been inserted. These nodes are referenced in the annotations element, which specifies the terms found in the text and their `Annotations`. For example, we see that the text between nodes 4 and 5, i.e. the first occurrence of *CRYG*, belongs to the term class 'gene' and that this gene has OMIM number 123730 and HUGO identifier (hgnc id) 2417.

The web service described above is complemented with a web-based GUI. The purpose of the GUI is to give the user an opportunity to assess the functionality of the web service without having to set up a web service client and engage in a full web service interaction. Through the GUI the user can submit a short text fragment for term look-up and inspect the results. As in the full web service, the user can select the classes of the terms that are to be looked up. The results of term look-up are presented in the format shown in Figure 2. In the tables appearing above the document text, each term class is assigned a colour. The terms occurring in the text are marked-up in the colours of the term classes to which they belong. The annotation viewer also displays any additional information that is associated with a term. Clicking on a line in the table of a particular term class will highlight the term occurrence in the text to which the information given in that line applies. Conversely, clicking on a term occurrence in the text will highlight the lines in the term class tables containing the information associated with this term.

### Implementation

Termino is implemented as a collection of processing modules which run within the GATE architecture [5,14]). The web service interface is implemented in Java, using the Apache Axis web services toolkit [1] running in the Apache Tomcat web server [2]. In order to get a first impression of the performance of the web service, we submitted 100 MEDLINE abstracts to the web service for processing. The average size of these abstracts was 1.1 kB. Processing the abstracts took 1 min 52 s, i.e. approximately 1.1 s/abstract. On average, 11.7 terms were marked-up in each abstract. The finite state recognizers compiled from Termino's database for use by the web service cover about 80,000 terms.

The browser-based interface is implemented as a Java servlet which makes use of the same processing modules as the web service to annotate the supplied text. The results are rendered using Java Server Pages (JSP) to generate an HTML page which uses a library of JavaScript functions to allow the user to explore the terms found by Termino. The web-based service can be found (at the time of writing) at **http://don.dcs.shef.ac.uk/**

Copyright © 2005 John Wiley & Sons, Ltd. 

```
<?xml version='1.0' encoding='UTF-8' ?>
<TaggedDocument>
<Text>Since mutations in the gamma-crystallin encoding
<Node id='4'/>CRYG<Node id='5'/> genes have previously been
demonstrated to be the most frequent reason for isolated
congenital <Node id='0'/>cataracts<Node id='1'/>, all 4
active
<Node id='2'/>CRYG<Node id='3'/> genes have been sequenced.
</Text>
<Annotations>
      <Gene startNode='2' endNode='3'>
            <omim_number>123730</omim_number>
      </Gene>
      <Gene startNode='4' endNode='5'>
            <hgnc_id>2417</hgnc_id>
      </Gene>
      <Gene startNode='4' endNode='5'>
            <omim_number>123730</omim_number>
      </Gene>
      <Gene startNode='2' endNode='3'>
            <hgnc_id>2417</hgnc_id>
      </Gene>
      <Disease startNode='0' endNode='1'>
            <cui>C0007388</cui>
      </Disease>
</Annotations>
</TaggedDocument>
```

**Figure 1.** Marked-up document

**termino/**, along with a WSDL definition for the web service interface.

### Conclusion

Termino is a large-scale terminological resource for biomedical text processing, developed to provide term recognition capabilities for information extraction, retrieval, and navigation. Biomedical term recognition is an essential part of the process of managing the wealth of biomedical information that is available electronically in structured databases and textual information sources.

Termino has been integrated into AMBIT, our platform for biomedical language processing [13]. Since access to Termino could be helpful to other research groups wanting to integrate a terminological resource into their applications, we have made Termino available as a web service. A web service provides public, standardized access to a resource over the web. The Termino web service delivers lexical look-up functionality: given a text, the service will return a version of the text in which term occurrences are identified and marked up with information from the Termino database.

The current prototype is available at **http://don.dcs.shef.ac.uk/termino/**. Future work will focus on extending the set of available term classes and their contents, as well as on adding further options for configuring the functionality of the web service so that users can request types of document mark-up that are specifically tailored to their biomedical needs.

### Acknowledgements

We would like to acknowledge the support of the UK Medical Research Council (Grant No. 60086) which has made possible the work reported here.

Copyright © 2005 John Wiley & Sons, Ltd. 

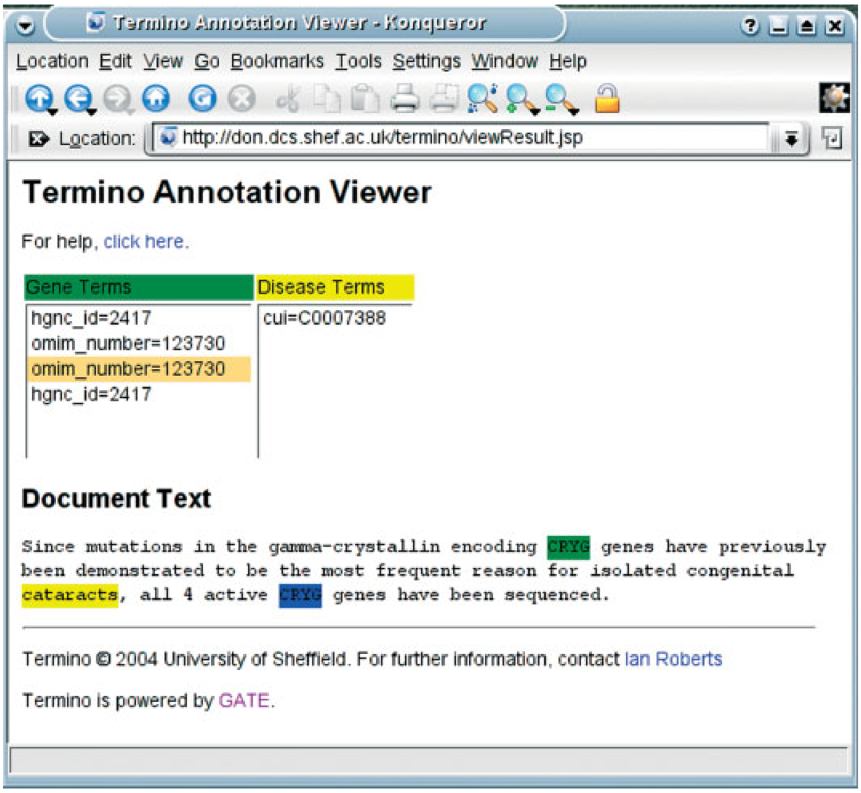

**Figure 2.** Screen shot of the web-based GUI

### References

1. Apache Axis web services toolkit; **http://ws.apache.org/axis/**
2. Apache Tomcat web server; **http://jakarta.apache.org/tomcat/**
3. Biemann C, Bordag S, Quasthoff U, Wolff C. 2004. Web services for language resources and language technology applications. In *Proceedings of the Fourth International Conference on Language Resources and Evaluation*, Lino MT, Xavier MF, Ferreira F, Costa R, Silva R (eds), Lisbon, Portugal.
4. Chen H, Sharp BM. 2004. Content-rich biological network constructed by mining PubMed abstracts. *BMC Bioinformat* **5**: 147; **http://www.biomedcentral.com/1471–2105/5/147**.
5. Cunningham H, Maynard D, Bontcheva K, Tablan V. 2002. GATE: a framework and graphical development environment for robust NLP tools and applications. In *Proceedings of the 40th Anniversary Meeting of the Association for Computational Linguistics*, Philadelphia, PA, USA.
6. Curran JR. 2003. Blueprint for a high performance NLP infrastructure. In *Proceedings of the HLT/NAACL Workshop on Software Engineering and Architecture of Language Technology Systems*, Edmonton, Canada.
7. Dalli A, Tablan V, Bontcheva K, *et al*. 2004. Web services architecture for language resources. In *Proceedings of the Fourth International Conference on Language Resources and Evaluation*, Lino MT, Xavier MF, Ferreira F, Costa R, Silva R (eds), Lisbon, Portugal.
8. European Bioinformatics Institute. Gene Ontology Annotation at EBI; **http://www.ebi.ac.uk/GOA/**
9. European Bioinformatics Institute, European Molecular Biology Laboratory. EMBL-EBI Web Services Home; **http://www.ebi.ac.uk/Tools/webservices/index.html**
10. Ferris C, Farrell J. 2003. What are web services? *Commun ACM* **46**(6): 31.
11. Gaizauskas R, Davis N, Demetriou G, Guo Y, Roberts I. 2004. Integrating text mining into distributed bioinformatics workflows: a web sevices implementation. In *Proceedings of the IEEE International Conference on Services Computing*, Shanghai, China.
12. Gaizauskas R, Demetriou G, Artymiuk P, Willett P. 2003. Protein structures and information extraction from biological texts: the PASTA system. *Bioinformatics* **19**(1): 135–143.
13. Gaizauskas R, Hepple M, Davis N, *et al*. 2003. AMBIT: acquiring medical and biological information from text. In *Proceedings of the UK e-Science All Hands Meeting*, Cox SJ (ed.), Nottingham, UK.
14. GATE — General Architecture for Text Engineering; **http://www.gate.ac.uk/**
15. Hahn U, Romacker M, Schulz S. 2002. Creating knowledge repositories from biomedical reports: the medSynDiKATe text mining system. In *Proc Pacific Symp Biocomput* **91**(2): 338–349.

Copyright © 2005 John Wiley & Sons, Ltd. 

16. Harkema H, Gaizauskas R, Hepple M, *et al*. 2004. A large-scale resource for storing and recognizing technical terminology. In *Proceedings of the Fourth International Conference on Language Resources and Evaluation*, Lino MT, Xavier MF, Ferreira F, Costa R, Silva R (eds), Lisbon, Portugal.
17. Harkema H, Gaizauskas R, Hepple M, *et al*. 2004. A large scale terminology resource for biomedical text processing. In *Proceedings of the HLT-NAACL Workshop: BioLINK 2004, Linking Biological Literature, Ontologies and Databases*, Boston, MA, USA.
18. Hirschman L, Morgan A, Yeh AS. 2002. Rutabaga by any other name: extracting biological names. *J Biomed Inform* **35**: 247–259.
19. Humphreys L, Lindberg DAB, Schoolman HM, Barnett GO. 1998. The Unified Medical Language System: an informatics research collaboration. *J Am Med Inform Assoc* **1**(5): 1–13.
20. McKusick–Nathans Institute for Genetic Medicine, Johns Hopkins University (Baltimore, MD) and National Center for Biotechnology Information, National Library of Medicine (Bethesda, MD). 2000. Online Mendelian inheritance in Man, OMIM (TM); **http://www.ncbi.nlm.nih.gov/omim/**
21. National Center for Biotechnology Information. Basic local alignment search tool (BLAST); **http://www.ncbi.nlm.nih.gov/BLAST/**
22. National Library of Medicine. MEDLINE; **http://www.ncbi.nlm.nih.gov/PubMed/**
23. National Library of Medicine. Unified Medical Language System; **http://www.nlm.nih.gov/research/umls/**
24. Quasthoff U, Wolff C. 2003. Web services in language technology and terminology management. In *Proceedings of the 6th Terminology in Advanced Management Applications Conference*, Pretoria, South Africa.
25. Swanson DR, Smalheiser NR. 1997. An interactive system for finding complementary literatures: a stimulus to scientific discovery. *Artif Intell* **91**: 183–203.
26. The Gene Ontology Consortium. 2001. Creating the Gene Ontology resource: design and implementation. *Genome Res* **11**(8): 1425–1433.
27. World Wide Web Consortium (W3C). Web services activity, 2004. **http://www.w3.org/2002/ws/**
28. Wain HM, Lush M, Ducluzeau F, Povey S. 2002. Genew: The human nomenclature database. *Nucleic Acids Res* **30**(1): 169–171.

Copyright © 2005 John Wiley & Sons, Ltd.
